# Comparative analysis of antibodies to SARS-CoV-2 between asymptomatic and convalescent patients

**DOI:** 10.1016/j.isci.2021.102489

**Published:** 2021-05-01

**Authors:** Connor J. Dwyer, Colleen A. Cloud, Cindy Wang, Philip Heidt, Paramita Chakraborty, Tara F. Duke, Shannon McGue, Braxton Jeffcoat, Jaclyn Dunne, Logan Johnson, Seungho Choi, Georges J. Nahhas, Amy S. Gandy, Nikolina Babic, Frederick S. Nolte, Philip Howe, Besim Ogretmen, Vamsi K. Gangaraju, Stephen Tomlinson, Brian Madden, Tracy Bridges, Patrick A. Flume, John Wrangle, Mark P. Rubinstein, Prabhakar K. Baliga, Satish N. Nadig, Shikhar Mehrotra

**Affiliations:** 1 Department of Surgery, Medical University of South Carolina, 86 Jonathan Lucas Street, Charleston, SC 29425, USA; 2 Department of Medicine, Medical University of South Carolina, 86 Jonathan Lucas Street, Charleston, SC 29425, USA; 3 Department of Psychiatry and Behavioral Sciences, Medical University of South Carolina, 86 Jonathan Lucas Street, Charleston, SC 29425, USA; 4 Clinical and Translational Research Center,Medical University of South Carolina, 86 Jonathan Lucas Street, Charleston, SC 29425, USA; 5 Laboratory and Pathology Medicine, Medical University of South Carolina, 86 Jonathan Lucas Street, Charleston, SC 29425, USA; 6 Biochemistry and Molecular Biology, Medical University of South Carolina, 86 Jonathan Lucas Street, Charleston, SC 29425, USA; 7 Microbiology & Immunology, Medical University of South Carolina, 86 Jonathan Lucas Street, Charleston, SC 29425, USA; 8 The Blood Connection, 1099 Bracken Road, Piedmont, SC 29673, USA

**Keywords:** Infection control in health technology, Immunology, Virology

## Abstract

The SARS-CoV-2 viral pandemic has induced a global health crisis, which requires more in-depth investigation into immunological responses to develop effective treatments and vaccines. To understand protective immunity against COVID-19, we screened over 60,000 asymptomatic individuals in the Southeastern United States for IgG antibody positivity against the viral Spike protein, and approximately 3% were positive. Of these 3%, individuals with the highest anti-S or anti-RBD IgG level showed a strong correlation with inhibition of ACE2 binding and cross-reactivity against non-SARS-CoV-2 coronavirus S-proteins. We also analyzed samples from 94 SARS-CoV-2 patients and compared them with those of asymptomatic individuals. SARS-CoV-2 symptomatic patients had decreased antibody responses, ACE2 binding inhibition, and antibody cross-reactivity. Our study shows that healthy individuals can mount robust immune responses against SARS-CoV-2 without symptoms. Furthermore, IgG antibody responses against S and RBD may correlate with high inhibition of ACE2 binding in individuals tested for SARS-CoV-2 infection or post vaccination.

## Highlights

• 60,000 asymptomatic individuals were assessed for SARS-CoV-2 antibody responses
• Increased Spike IgG correlates with antibody diversity and ACE2 binding inhibition
• COVID-19 patients had lower antibody responses compared with high asymptomatic responders
• Individuals can mount diverse immune responses to COVID-19 without symptoms

## Graphical abstract

**Figure:**
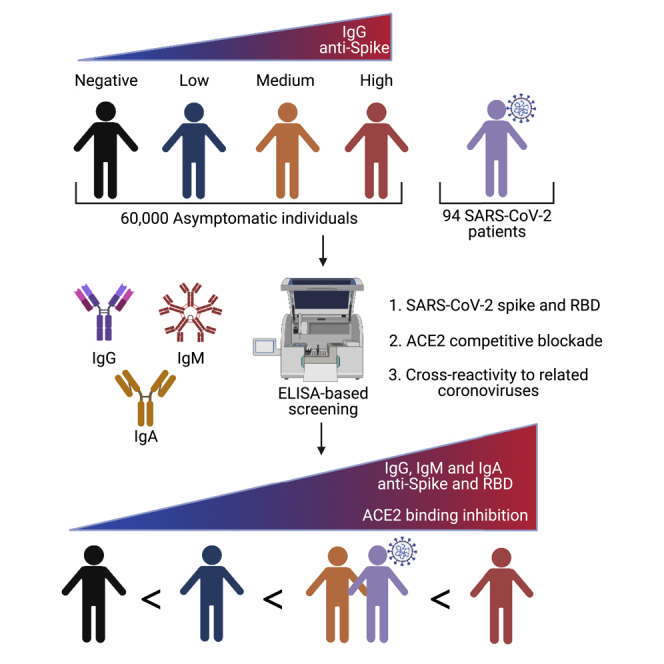

## Introduction

The new coronavirus (severe acute respiratory syndrome coronavirus 2 [SARS-CoV-2]) outbreak has spread to dangerous levels with a high level of morbidity in the United States and has created an urgent national health emergency (Ciotti et al., 2019; Hu et al., 2021). Clinical research is focused on understanding the mechanisms underlying viral infection and measures to reduce disease severity (Felsenstein et al., 2020; Lega et al., 2020; Ye et al., 2020). However, it is equally important to determine the degree of infection prevalent in the broader community and establish any unique phenotypes associated with asymptomatic individuals that render them less susceptible to disease. Thus, a detailed parallel comparison of the humoral and cellular immunity induced after SARS-CoV-2 infection in asymptomatic versus symptomatic individuals is needed (Amanat and Krammer, 2020; Sette and Crotty, 2021; Vabret et al., 2020).

The two primary antigenic targets of the SARS-CoV-2 virus against which antibodies are detected are Spike glycoprotein (S) and nucleocapsid phosphoprotein (N). S protein is essential for viral entry and is present on the viral surface, whereas N protein is the most abundantly expressed immunodominant protein that interacts with RNA. Multiple forms of S protein—full length (S1 + S2) or partial (S1 domain or receptor-binding domain [RBD])—are used as antigens for immunological assessment. Protein target expression determines cross-reactivity, with N being more conserved across coronaviruses than S, and within S, S2 and RBD are more conserved than S1 (Amanat and Krammer, 2020; Huang et al., 2021; Jaimes et al., 2020; Krammer and Simon, 2020). Serologic assays are critical tools for determining the prevalence of infection and immune responses to the virus and vaccines (Amanat et al., 2020; Long et al., 2020; Stadlbauer et al., 2020). Using sero-assays also helps estimate how much of the population is uninfected, allowing public health officials to plan future health care needs. Serology testing can provide information on the degree of infection in different geographical locations and ethnicities. Besides, sero-assays also serve as a screening tool for those who can donate therapeutic convalescent plasma (Duan et al., 2020) or monitor antibody levels upon vaccination.

To facilitate the development of vaccines against SARS-CoV-2, research efforts need to assess the level of antibodies generated after primary infection, the duration of antibody response, and if the response is protective against reinfection (Krammer, 2020). Additionally, the factors associated with the development of a protective antibody response—including its kinetics, the correlation of binding antibody titers to neutralization, and the protective titer of neutralizing antibodies—are yet to be determined. As a means to understand the antibody responses to SARS-CoV-2 in the general population, we developed an orthogonal ELISA-based sero-assay to perform large-scale antibody testing based on a previously reported protocol (Xu et al., 2020). We aim to determine the quality and quantity of the humoral immune response in asymptomatic individuals, providing protection and preventing the development of symptoms against SARS-CoV-2.

## Results

### Analysis of SARS-COV-2-infected asymptomatic individuals in the Southeastern United States

**Figure 1:**
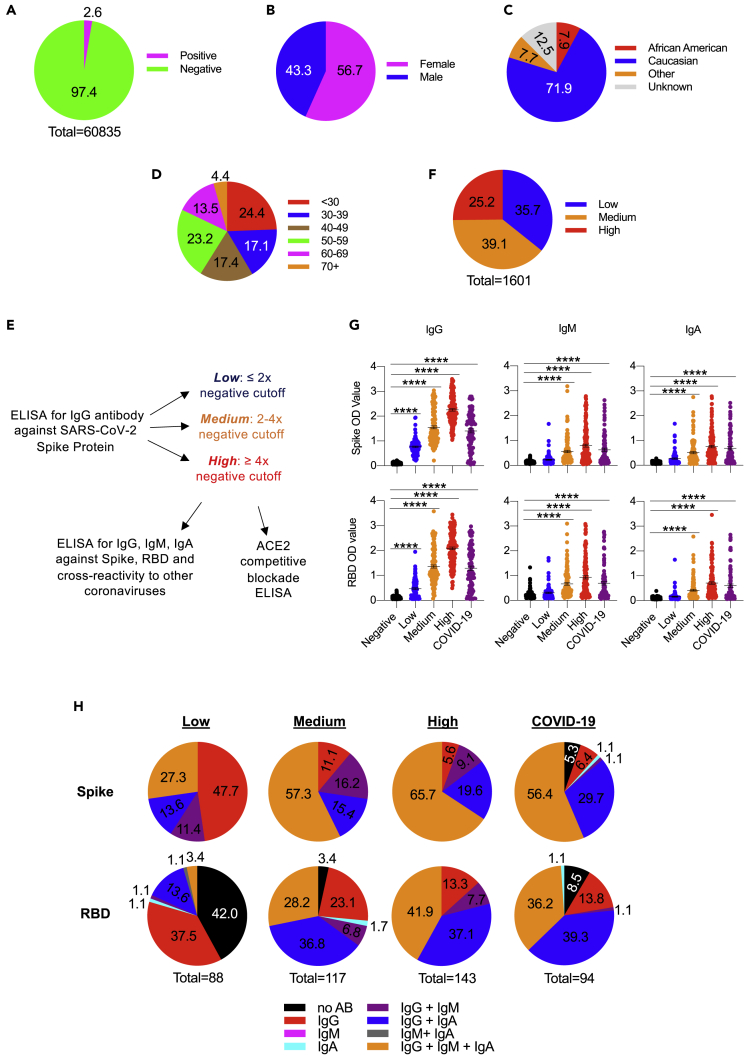
Serum antibody analysis of asymptomatic individuals (A–D) (A) Pie chart highlighting the fraction of asymptomatic positives out of 60,000 samples evaluated for ELISA-based anti-S IgG antibody levels. Pie charts of the positive samples depicting the distribution of gender (B), ethnicity/race (C), and age (D). (E) Workflow schematic for the analysis of asymptomatic positive samples. (F) Pie chart showing the distribution of positive samples based on OD values observed for anti-S IgG. *Low*: < twice the cutoff, *Medium*: 2−4 times the cutoff, *High*: > 4 times the cutoff. (G) Serum samples were analyzed for IgG, IgM, and IgA antibodies against SARS-CoV-2 Spike and receptor-binding domain using ELISA in *Negative* (n = 143), *Low* (n = 88), *Medium* (n = 117), *High* (n = 143), and SARS-CoV-2-infected patients seen at the MUSC hospital (n = 94). Each dot represents a sample within the group. Data are represented as the mean ± standard error of the mean. Data were analyzed by one-way ANOVA with Tukey's multiple comparisons test. ∗∗∗∗*p*-value <0.0001. (H) Pie charts showing the proportion of samples that expressed one, two, or three antibody subtypes in all categories of samples presented in (G).

To ascertain the percent of SARS-CoV-2-infected asymptomatic individuals within three southeastern states (SC, NC, GA), we initiated a large-scale antibody testing program at MUSC using ELISA-based testing (Amanat et al., 2020; Stadlbauer et al., 2020), for which Emergency Use Authorization was submitted to the US Food and Drug Administration in May 2020. Our comprehensive data show less than 3% seropositivity in the community in the samples collected between May and July 2020 (Figure 1A). Of the positive population, 57% were female and 43% were male, and most were Caucasian (Figures 1B and 1C). Further analysis based on the ethnicity groups showed that antibody positivity was relatively higher in African Americans than Caucasians (Table 1). Similarly, the individuals younger than 30 years showed higher antibody prevalence (Figure 1D). There was a broad age distribution, with the largest groups being less than 30 years old and individuals 50–59 years old.

**Table 1:** SARS-CoV-2 asymptomatic sample demographics

	% Of Total	% Of Positive	% Of Negative
**Gender**			

Female	55.8	56.7	55.8
Male	44.2	43.3	44.2

**Race/Ethnicity**			

African American	4.2	7.9	4.1
Dravidic (India, Pakistan)	0.3	0.2	0.3
Indian/Alaska native	0.1	0	0.1
Latino/Hispanic	2.4	4.8	2.3
Mediterranean	0.1	0.2	0.1
Multiracial	0.3	0.4	0.3
Native American/Alaska native	0.1	0.1	0.1
Native Hawaiian/Pacific Islander/Melanesian	0.1	0.2	0.1
Asian	1	1.6	0.9
White	81.5	71.9	81.7
Unknown	10.2	12.5	10.1

**Age**			

<30	16.8	24.4	16.6
30–39	18	17.1	18.1
40–49	19.9	17.4	20
50–59	22.7	23.2	22.7
60–69	16.7	13.5	16.7
70+	5.8	4.4	5.9

### Comparable SARS-CoV2 antibodies in asymptomatic individual and convalescent patient serum

For this analysis, individuals who did not exhibit any symptoms when providing samples through The Blood Connection but had positive antibody response were considered asymptomatic infected individuals and were compared against the convalescent plasma samples (n = 94) from the MUSC coronavirus disease 2019 (COVID-19) biorepository collected from hospitalized patients with COVID-19 symptoms. A comprehensive evaluation of the antibody subtypes (IgG, IgM, IgA) was performed. Additionally, we used a cPass neutralization antibody detection kit (GenScript), which determines the circulating neutralizing antibodies against SARS-CoV-2 based on its ability to block the interaction between the RBD of the viral Spike glycoprotein with the ACE2 cell surface receptor (Tan et al., 2020). We observed that the samples from asymptomatic individuals and the convalescent patient serum exhibit comparable levels of IgG antibodies against SARS-CoV-2 S and RBD (Figure S1A). The levels of IgM and IgA were lower, whereas they were similar in both asymptomatic positives and convalescent individuals. An analysis determining the percent positive samples for one, two, or all three antibody subtypes showed that about 50% of both asymptomatic and convalescent individuals have all three subtypes (IgG + IgA + IgM) of antibodies against S protein (Figure S1B*, upper panel*). However, 30% of each sample type has double (IgG + IgA) and the same percentage has all three (IgG + IgA + IgM) against RBD protein (Figure S1B*, lower panel*).

Interestingly, the level of antibodies detected for the other coronavirus-related proteins (MERS-CoV, SARS-CoV, hCoV-HKU1, hCoV-OC43) was higher in asymptomatic patients than in convalescent patient serum (Figure S1C). However, we noticed relatively lower IgG and IgA levels for hCoV-NL63 in the serum of asymptomatic positive patients. Notably, using the ACE2 competitive blockade assay as a surrogate for serum neutralizing antibodies, inhibition in both groups was significantly higher than the negative samples for SARS-CoV-2. The convalescent patient samples showed a slightly increased median value of inhibition of ACE2 binding compared with the other groups (Figure S2A). Regression analysis showed that anti-S and anti-RBD IgG levels were strongly correlated with ACE2 blockade, more so than the IgM and IgA levels (Figures S2B–S2D).

### Low antibody response in hospitalized patients compared with asymptomatic *high* responders

Given a wide range of antibody levels in the positives over the cutoff, we segregated the data from the asymptomatic individuals into three groups based on the optical density (OD) values for anti-S antibody levels. Those with OD values within twice the cutoff were assigned *Low*, whereas those with OD values between 2−4 times the cutoff were set as *a Medium* and those with OD values above 4 times the cutoff were designated as *High* (Figure 1E). The percent distribution for *High* was 25%, whereas values for *Medium* and *Low* were 39% and 36%, respectively (Figure 1F).

Data from individuals with high anti-S and anti-RBD IgG levels also exhibited high levels of IgM and IgA antibodies (Figure 1G). Notably, all antibody subtypes (IgG, IgM, IgA) were lower in convalescent patient serums than the antibody levels found in the *High* category of asymptomatic individuals. The levels of antibody subtypes in convalescent patients were closer to the ones found in the *Medium* category of asymptomatic individuals (Figure 1G). The *Low* asymptomatic individuals predominantly had the highest anti-S and anti-RBD IgG (Figure 1H, left pie chart, red fraction). However, similar to convalescent patients, the *Medium* and *High* category samples exhibited the highest levels of all three isotypes (IgG + IgA + IgM). Notably, this fraction was highest in *the High* group (66%), followed by *the Medium* group (57%) and convalescent samples (56%). All three subtype anti-RBD antibodies were predominant in the *High* sample group (42%), whereas *Medium* and convalescent groups showed high levels of only two subtypes (IgG + IgA). These data indicate that antibody levels and subtypes observed in the convalescent group were similar to those observed in the *Medium* asymptomatic individuals (with anti-S IgG 2−4 times the cutoff).

**Figure 2:**
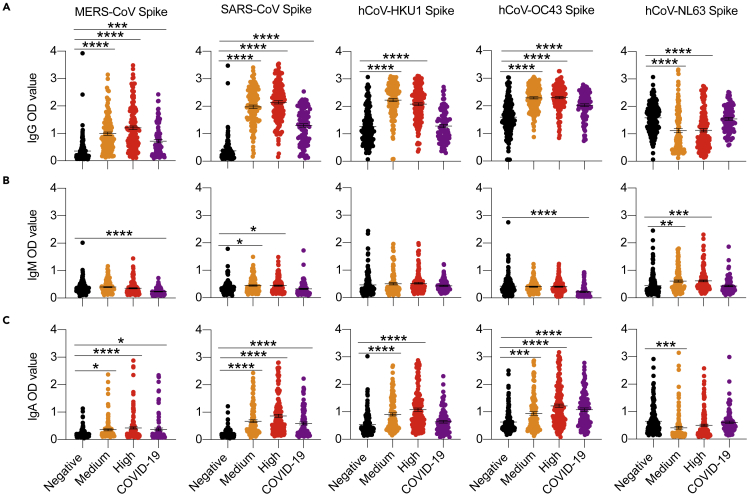
Cross-reactive antibody analysis of asymptomatic and convalescent individuals (A−C) Serum samples from individuals positive for COVID-19-specific IgG antibodies were used to determine the circulating antibodies against other related coronaviruses. ELISA-based analysis of serum antibodies against non-COVID coronavirus Spike proteins for IgG (A), IgM (B), and IgA (C) in asymptomatic positives from *Medium* and *High* groups, negatives, and COVD-19 patients. Data are represented as the mean ± SEM. One-way ANOVA analyzed data with Tukey's multiple comparisons test. ∗p<0.05, ∗∗p<0.01, ∗∗∗p<0.001, ∗∗∗∗p<0.0001.

We also noted that the anti-IgG antibody levels for the related coronaviruses were enhanced in asymptomatic individuals in the *High* and *Medium* categories, except that convalescent patients had higher hCoV-NL63 IgG levels (Figure 2A). The levels of IgM and IgA for other coronavirus proteins did not differ much between groups (Figures 2B and 2C).

### Positive correlation between antibody levels and the inhibition of ACE2 binding

**Figure 3:**
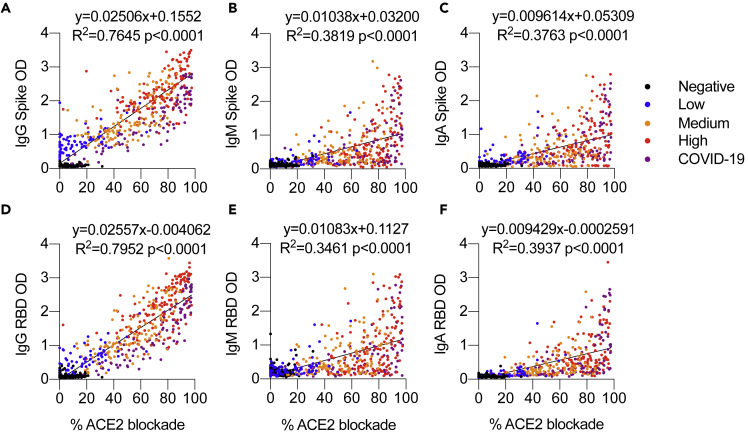
Inhibition of ACE2 binding positively correlates with IgG antibodies reactive to Spike or RBD (A−F) Serum IgG, IgM, and IgA reactive to Spike or RBD and percent ACE2 blockade from different groups of anti-S/RBD IgG-positive asymptomatic individuals and COVID-19 patients analyzed by ELISA. IgG Spike (A), IgM Spike (B), IgA Spike (C), IgG RBD (D), IgM RBD (E), and IgA RBD (F). Data were analyzed by nonlinear regression and two-tailed correlation analysis between percent ACE2 blockade with IgG, IgM, and IgA.

As the presence of a non-neutralizing antibody response to conserved epitopes in the Spike protein has been shown (Lv et al., 2020), we determined if there is any correlation between the anti-S and anti-RBD antibodies to the inhibition of ACE2 binding. Correlation analysis between the IgG response and the ACE2 blockade showed that asymptomatic individuals with low anti-S IgG have lower ACE2 blockade and high anti-S/RBD IgG antibodies likewise have higher levels of ACE2 blockade (Figure 3). These data suggest that a simple anti-S and anti-RBD IgG antibody determination could be valuable in predicting the antibodies with ability to inhibit ACE2 binding in any individual. Similarly, the levels of anti-S/RBD IgG in the patient samples ranged from low to high; the ACE2 binding inhibition levels also showed an equally broad range (Figure 3).

### Higher antibody levels in patients in the ICU compared with those in outpatient and inpatient settings

**Figure 4:**
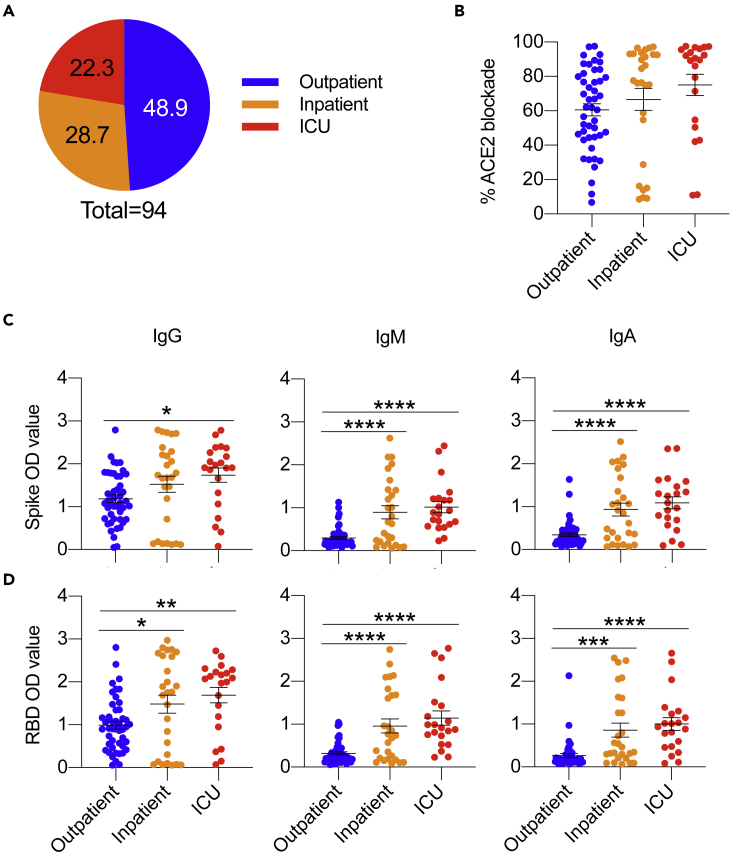
Inpatients in the ICU have increased antibodies reactive to Spike and RBD proteins (A) Pie chart highlighting the fraction of SARS-CoV-2-infected patients seen at the MUSC hospital as outpatients (n = 46), inpatients (n = 27), and inpatient ICU (n = 21). (B–D) (B) SARS-CoV-2-infected patient serum analyzing percent ACE2 blockade. SARS-CoV-2-infected patient serum samples were analyzed for IgG, IgM, and IgA antibodies against SARS-CoV-2 Spike (C) and RBD (D) using ELISA. Data are represented as the mean ± SEM. One-way ANOVA analyzed data with Tukey's multiple comparisons test. ∗p<0.05, ∗∗p<0.01, ∗∗∗p<0.001, ∗∗∗∗p<0.0001.

To further analyze the antibody levels of COVID-19 patients, samples were grouped based on clinical grounds (outpatient, inpatient, or intensive care unit [ICU]) (Figure S3A). In the 94-patient cohort, 50% were outpatient, whereas inpatient and ICU made up the other half (Figure 4A). In the clinical categories, each group had a similar distribution of males and females, and each group was predominantly Caucasian with a small proportion of African American and Hispanic individuals. The outpatient cohort was skewed toward younger individuals (20–44 years of age), whereas the inpatient and ICU cohorts had more individuals aged 45–94 years (Figure S3A). Analysis of the COVID-19 patients by ethnicity, age, and sex showed minor changes in ACE2 competitive inhibition and Spike and RBD antibody response (Figures S3B–S3E). ACE2 competitive inhibition analysis showed an increasing trend of activity for patiemts in the ICU (Figure 4B). IgG, IgM, and IgA antibody subtypes for anti-S and anti-RBD were highest among the patients in the ICU (Figures 4C and 4D). Insignificant differences were observed in cross-reactivity to non-COVID-19 spike proteins (MERS-CoV, SARS-CoV, hCoV-HKU1, hCoV-OC43) between patient groups except for significantly decreased IgG antibodies toward hCoV-NL63 in the patients who were admitted to the ICU (Figure S4).

## Discussion

COVID-19 is an ongoing global pandemic caused by SARS-CoV-2. For public health control of the virus, it will be vital to have a strategy for categorizing individuals based on their immune response to the virus. A comprehensive characterization of the immune response to SARS-CoV-2 infection can help minimize its further spread, ascertain seroprevalence, and direct therapeutic strategies (Kellam and Barclay, 2020; Krammer, 2020; Sette and Crotty, 2020). Much is yet to be determined regarding serologic antibody testing, including the kinetics of the antibody response, longevity of antibodies, antibodies' ability to protect from repeat infection, the protective titer of neutralizing antibody, and the correlation of binding antibody titers to neutralization ability (Klasse and Moore, 2020; Netea et al., 2020). Population antibody testing is key to obtaining such data. We initiated and established a large-scale antibody testing program and performed more than 60,000 antibody tests to determine IgG antibodies against S protein in the community serum samples.

Our data show 2.63% seropositivity in the community; we also observed that individuals 30 years or younger showed the highest antibody response compared with others. Further analysis based on the ethnicity groups showed that antibody positivity was higher in African Americans. These findings are concordant with previously published reports indicating that COVID-19 cases are disproportionately high in young individuals and African Americans in southeastern states of SC, NC, and GA. Identifying groups with higher disease prevalence will help public health leaders target interventions and prioritize resources.

It is well known that sampling time may influence humoral profiles, and could compare immature versus mature immune responses. Despite the group's sampling differences, comparable titers were observed across convalescents and asymptomatic individuals in the *Medium* or *High* cohort. We observed that IgG, IgM, and IgA were all present at high titers in asymptomatic *High* responders and convalescent patient samples. Interpreting antibody response patterns has been difficult because of ambiguity about when antibodies develop and wane in the course of infection.

A recent longitudinal study of COVID-19 patients showed that three seroconversion patterns occurred with approximately equal frequency: IgM earlier than IgG seroconversion, IgG earlier than IgM seroconversion, and simultaneous IgM and IgG seroconversion (Long et al., 2020; Ma et al., 2020). Our findings that all three antibodies are present at similar titers in symptomatic patients and asymptomatic *High* responders suggest that the asynchrony between antibody subtype appearance is minimal. IgA also develops in individuals infected with SARS-CoV-2 (Guo et al., 2020) and is essential for mucosal immunity. A limitation of our study is the lack of follow-up sampling from our asymptomatic or clinical cohorts. Antibody responses to Spike and RBD (IgG, IgM, and IgA) and neutralizing antibodies have all been reported to decrease rapidly after SARS-Cov-2 resolution (Beaudoin-Bussières et al., 2020; Gaebler et al., 2020; Muecksch et al., 2021; Prévost et al., 2020). As the B cell compartment is heavily shaped by SARS-CoV-2 infection, understanding the difference in protective antibody dynamics in asymptomatic and clinical cohorts may identify fundamental mechanisms to prevent symptom manifestation (Gaebler et al., 2020). A recent longitudinal study assessing individuals who recovered from mild COVID-19 showed that development of SARS-CoV-2-specific immunoglobulin (IgG) antibodies, neutralizing plasma, and memory B and memory T cells not only persisted for at least 3 months but also increased over time (Rodda et al., 2021). Similarly, another study showed that IgG to the Spike protein was relatively stable over 6 months and that Spike-specific memory B cells were more abundant at 6 months than at 1 month post symptom onset (Dan et al., 2021).

We also detected significant cross-reactivity of the antibody response in the asymptomatic individuals than the convalescent patients, similar to recent reports (Beaudoin-Bussières et al., 2020; Lv et al., 2020; Prévost et al., 2020). Even though previous coronavirus outbreaks were less widespread geographically, detecting antibodies cross-reactive to Spike proteins for those viruses may have contributed to the reduced severity in the asymptomatic individuals. Another explanation of the increased cross-reactivity is due to the high conservation of S2 between MERS-CoV, SARS-CoV, hCoV-HKU1, and hCoV-OC43, which are related betacoronaviruses (Huang et al., 2021; Jaimes et al., 2020). Increased antibody production against SARS-CoV-2 could lead to cross-reactivity in the *High* and *Medium* cohorts, which have the highest SARS-CoV-2 antibody levels.

In contrast to studies conducted by Beaudoin-Bussières et al. and Prévost et al., our *Medium* and *High* responder groups had detectable but decreased IgG antibodies toward hCoV-NL63 compared with convalescent patients. These studies had no detectable NL63 antibody response. Decreased hCoV-NL63 antibodies may indicate that immune responses to such previous antigens may have resulted in “immune-enhancement” (Arvin et al., 2020) and the increased severity of the disease. Further comprehensive analysis needs to be done to establish if the disease severity to SARS-CoV2 exists due to a phenomenon observed in many other infections caused by closely related viruses termed “original antigenic sin” (Fierz and Walz, 2020).

Analyzing neutralizing antibodies in all infected patients or asymptomatic individuals is logistically challenging. Based on our data, we believe that anti-S IgG's presence using routine ELISA-based tests can help ascertain the possibility of neutralizing antibodies in any individual. Recent reports showed that symptom severity positively correlates with enhanced neutralizing antibodies and levels of anti-Spike and RBD IgG, IgM, and IgA (Chen et al., 2020b; Gaebler et al., 2020; Garcia-Beltran et al., 2021; Seow et al., 2020). We show that *High* asymptomatic individuals have higher inhibition of ACE2 binding and anti-Spike and anti-RBD responses than our hospitalized patient cohort. This observation is intriguing as several studies have shown minimal responses in healthy asymptomatic controls. These results highlight the possibility of an innate ability of asymptomatic immune systems to control high viral loads and mount robust adaptive immunity without developing symptoms.

It has been elegantly demonstrated earlier (Buchholz et al., 2004) that the Spike glycoprotein is the sole structural protein of SARS-CoV, which is necessary and sufficient to induce a neutralizing antibody response and protection from the challenge (Buchholz et al., 2004). Recent studies also demonstrated that anti-S IgG levels were higher in the patients who recovered than those who were deceased (Chandrashekar et al., 2020). However, SARS-CoV-2 anti-RBD antibodies with potent neutralizing activity have also been found in individuals with moderate plasma neutralizing activity (Duan et al., 2020). The potential role of S-targeted immunity in viral control is also highlighted in new studies in non-human primates, demonstrating elevated and robust functional humoral immune responses to S, rather than RBD and N, following primary infection that were associated with protection upon re-exposure to the virus (Chandrashekar et al., 2020). Another recent study showed that the Spike ELISA endpoint titers correlated with viral neutralization (Salazar et al., 2020). Thus, a strong correlation between anti-S IgG and inhibition of ACE2 binding, as observed in this study, indicates that vaccines inducing antibodies to whole S protein or RBD of SARS-CoV-2 may effectively control disease severity.

An essential aspect of the data presented here is that upon segregation based on the anti-S IgG levels, we found that serum inhibition of ACE2 binding was highest in the group with OD values more than 4 times the cutoff. The trend declined with reduced OD vales (i.e., *Medium* to *Low*, and none in negative samples). As the COVID-19 patient samples had lower levels of ACE2 binding inhibition compared with the *High* group and more similar to the *Medium* cohort, it appears that even the asymptomatic individuals can develop robust immunity. However, with the recent reports showing a decline in the antibody titers within 2−3 months (Seow et al., 2020), it will be essential to have a longitudinal follow-up of the asymptomatic individuals to establish a similar quantitative decrease in antibody response as reported with COVID-19 patients. With the administration of several COVID-19 vaccines, our serologic assay could be purposed to follow longitudinal immune responses to the vaccine. In addition to following neutralizing antibodies to measure vaccine efficacy, our assay could compare how individuals respond to the same vaccine measuring the diversity of antibody responses. Once the relationship between antibody detection and protective immunity is clarified, our results could also be used to stratify individuals by infection risk, identify individuals who would require vaccine boosters, and identify potential donors with high anti-S IgG (and thereby ability to inhibit ACE2 binding) for convalescent plasma, which has been shown to improve outcomes in critically ill COVID-19 patients (Chen et al., 2020a). Beyond clinical medicine, serologic tests could have essential roles in public health by characterizing COVID-19 disease burden, the prevalence of asymptomatic infections, case mortality, and level of herd immunity.

### Limitation of the study

In this study, we assessed more than 60,000 asymptomatic individuals who provided samples through The Blood connection and a small patient cohort at MUSC for antibody responses to Spike and RBD. This study was limited due to a lack of longitudinal follow-up for the samples from our symptomatic or patient cohorts over time and the use of the cPass neutralization antibody detection kit. Over time, assessment of these cohorts may have provided differences in SARS-CoV-2 antibody dynamics between asymptomatic individuals and patients. However, we can stratify our results on our patient cohort based on disease severity, age, sex, and ethnicity; information regarding the time since symptom onset was not available to us. As SARS-CoV-2 antibody responses have been shown to decrease with viral resolution and time, this was a limiting factor of our data (Beaudoin-Bussières et al., 2020; Gaebler et al., 2020; Muecksch et al., 2021; Prévost et al., 2020). Additionally, as the cPass neutralization antibody detection kit measures competitive inhibition of ACE2 binding rather than direct neutralization activity, we were unable to demonstrate neutralizing activity of the serum samples.

## STAR★methods

### Key resources table

**Table:** 

REAGENT or RESOURCE	SOURCE	IDENTIFIER
**Antibodies**		

Goat anti-human IgA cross-adsorbed secondary antibody, HRP	Invitrogen	Cat# A18787;RRID:AB_2535564
Goat anti-human IgM secondary antibody, HRP	Invitrogen	Cat# 31415; RRID:AB_228282
Goat anti-human IgG Fc, multi-species SP ads-HRP	SouthernBiotech	Cat# 2014-05; RRID:AB_2795580

**Biological samples**		

Asymptomatic individual serum	The Blood Connection	N/A
COVID-19 patient serum	MUSC Biorepository	N/A

**Chemicals, peptides, and recombinant proteins**		

SARS-CoV-2 Spike protein	LakePharma	Cat# 46328
SARS-CoV-2 RBD protein	LakePharma	Cat# 46438
MERS-CoV Spike protein	Sino Biological	Cat# 40069-V08B
SARS-CoV Spike protein	Sino Biological	Cat# 40634-V08B
hCoV-HKU1 Spike protein	Sino Biological	Cat# 40606-V08B
hCoV-OC43 Spike protein	Sino Biological	Cat# 40607-V08B
hCoV-NL63 Spike protein	Sino Biological	Cat# 40604-V08B

**Critical commercial assays**		

SARS-Cov-2 surrogate virus neutralization test	Genscript	Cat# L00847

### Resource availability

#### Lead contact

Further information and requests for resources and reagents should be directed to and will be fulfilled by the lead contact, Shikhar Mehrotra (mehrotr@musc.edu).

#### Material availability

This study did not generate new unique reagents.

#### Data and code availability

This study did not generate new datasets.

### Experimental model and subject details

All samples for development and validation of methods were collected from apparently healthy volunteers or de-identified residual clinical samples. The general population samples were obtained through The Blood Connection, a community blood center in the Southeast. Samples were collected from 687 zip codes from states of South Carolina (534 zip codes, 46 counties), North Carolina (133 zip codes, 56 counties), and GA (20 zip codes, 15 counties). Asymptomatic samples were obtained from both males and females ages 16 to 83. After collection, samples were shipped over-night to the MUSC’s COVID-19 serology test site for performing the ELISA. SARS-CoV-2 infected patient samples banked at MUSCs COVID-19 biorepository were obtained after appropriate IRB approvals. Patient samples were collected from both males and females ages 20 to 94.

### Method details

#### Enzyme-linked immunosorbent assay (ELISA)

To validate our ELISA assay, antibodies binding to the virus's surface glycoproteins - S protein and RBD - were determined using the serological assay described earlier (Stadlbauer et al., 2020). The 96 well microtiter plates were coated overnight at 4°C using the commercially available S-protein (LakePharma, Cat # 46,328) or RBD-protein (LakePharma Cat # 46,438) at 2 μg/mL. Plates were then washed thrice and blocked for one hour with PBS-Tween + 3% milk powder (weight/volume). The samples were diluted at 1:200, added to each well, and incubated for 2 hr. The plates were washed, and anti-human IgG (Fab specific) HRP labeled secondary antibody (SouthernBiotech, Cat# 2014-15) 1:3000 in PBS-T containing 1% milk was added for 1 hr. Subsequently, plates were washed, and substrate (SIGMA*FAST* OPD; Sigma-Aldrich) was added. The reaction was stopped by adding 50 μL of 3 M HCl to all wells before reading at 490 nm to record the data. Inactivated human AB serum stored from pre-COVID times was used as a negative control, while monoclonal antibody CR3022 was used as a positive control to ensure assay reproducibility.

Precision was tested using negative QC and positive QC material. Quadruple replicates were performed on each QC level within two runs using both RBD and S data. Additionally, the specificity of the ELISA assay was validated by testing serum and plasma positive for non-SARS-CoV-2 viruses (Table S1). Control limits were defined using the samples from pre-pandemic, drawn more than a year ago, known as true negatives. The average value of the actual negative samples plus three standard deviations cut-off for a negative result will be an OD 490 of 0.45. For the SARS-CoV-2 Spike and RBD protein ELISA, a value of 0.45 or less was interpreted as negative, and a value of 0.45 or higher was interpreted as a positive result. Using this procedure, we performed >60,000 tests and handled approximately 1000 tests per day.

Following standardization, the positive asymptomatic samples with anti-S antibody above the cut-off were further categorized to *Low, Medium,* and *High* based on OD values (where *Low*: ≤ twice the cut-off, *Medium*: between two-four times the cut-off, and *High*: greater than four-times the cut-off). ELISA was then used to analyze all samples for IgG, IgM, and IgA reactive to Spike, RBD, or non-COVID-related spike proteins using anti-human IgG, anti-human IgM (Invitrogen, Cat# 31,415) and anti-human IgA (Invitrogen, Cat# A18787) HRP labeled secondary antibodies 1:3000 in PBS-T containing 1% milk. For cross-reactivity determination, the 96 well microtiter plates were coated overnight at 4°C using the commercially available proteins against MERS-CoV (Sino Biological Cat# 40,069-V08B), SARS-CoV (Sino Biological Cat# 40,634-V08B), hCoV-HKU1 (Sino Biological Cat# 40,606-V08B), hCoV-OC43 (Sino Biological Cat# 40,607-V08B), and hCoV-NL63 (Sino Biological Cat# 40,604-V08B) at 1.5ug/mL.

#### ACE2 competitive blockade assay

ACE2 competitive blockade was analyzed using the SARS-Cov-2 surrogate virus neutralization test reported recently (Tan et al., 2020). Serum was diluted 1:9 and then incubated 1:1 with HRP-RBD solution at 37°C for 30 min. Samples were then added to the human angiotensin-converting enzyme-2 (ACE2) coated plate (GenScript Cat# L00847) and incubated at 37°C for 15 min. Plates were washed four times with wash solution and developed with the addition TMB solution at room temperature for 15 min. Reactions were stopped with the provided stop solution, and results were read at 450 nm. Data were calculated using the provided positive and negative controls run in triplicate and the equation:

$$inhibition=\left(1-\left(\frac{sample\;OD\;value}{negative\;control\;OD\;value}\right)\right)x100$$

Values below 20% inhibition were considered negative per the kit's cut-off interpretation.

### Quantification and statistical analysis

One-way ANOVA analyzed ACE2 competitive blockade data with Tukey's multiple comparisons test. ELISA antibody data for IgG, IgM, and IgA reactive to S, RBD, or non-COVID-19 related S proteins were analyzed by one-way ANOVA with Tukey's multiple comparisons test or two-tailed unpaired T test. Correlation analysis between ACE2 blockade and IgG, IgM, or IgA reactive to S or RBD was analyzed using nonlinear regression analysis and two-tailed correlation analysis assuming Gaussian distribution. Statistical significance was defined as p value ∗<0.05, ∗∗<0.01, ∗∗∗<0.001, ∗∗∗∗<0.0001. Asymptomatic sample cohort sample size: *Negative* (n = 143), *Low* (n = 88), *Medium* (n = 117), *High* (n = 143). COVID-19 patient cohort sample size: Outpatient (n = 46), Inpatient (n = 27) and ICU (n = 21). For data comparing bulk groups: Positive (*Low* + *Medium* + *High*) n = 348 and COVID-19 (Outpatient + Inpatient + ICU) n = 94.
