# Supplementary material for: Comparative analysis of antibodies to SARS-CoV-2 between asymptomatic and convalescent patients
Source: iScience. 2021 May 1;24(6):102489. doi: 10.1016/j.isci.2021.102489 (PMC8087581; doi:10.1016/j.isci.2021.102489)
Supplement: Document S1. Figures S1–S4 and Table S1 [file mmc1.pdf]

iScience, Volume *24*

Supplemental information

Comparative analysis of antibodies to SARS-CoV-2 between asymptomatic and convalescent patients

Connor J. Dwyer, Colleen A. Cloud, Cindy Wang, Philip Heidt, Paramita Chakraborty, Tara F. Duke, Shannon McGue, Braxton Jeffcoat, Jaclyn Dunne, Logan Johnson, Seungho Choi, Georges J. Nahhas, Amy S. Gandy, Nikolina Babic, Frederick S. Nolte, Philip Howe, Besim Ogretmen, Vamsi K. Gangaraju, Stephen Tomlinson, Brian Madden, Tracy Bridges, Patrick A. Flume, John Wrangle, Mark P. Rubinstein, Prabhakar K. Baliga, Satish N. Nadig, and Shikhar Mehrotra

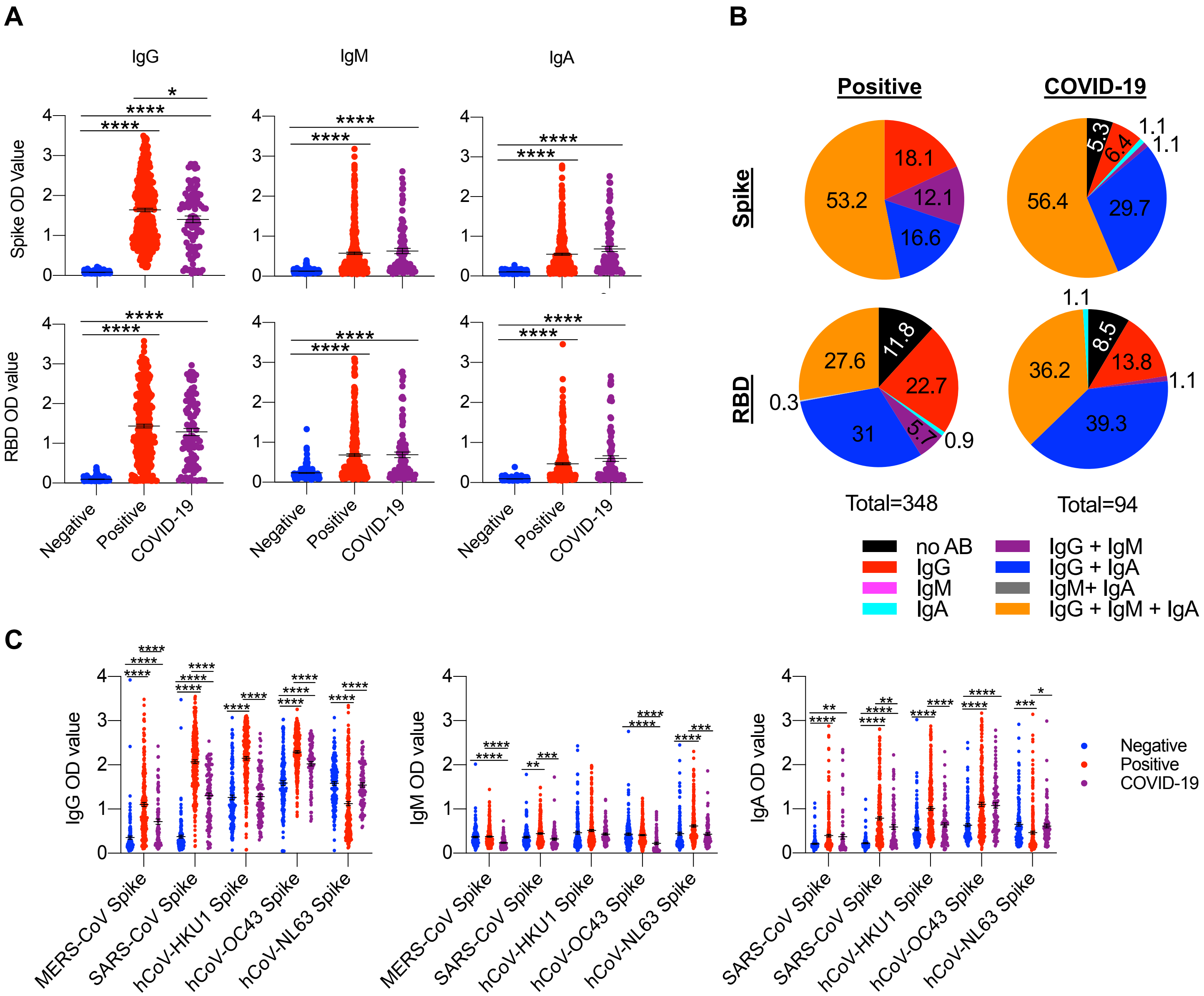

Figure S1. Related to Figure 1. **Antibody subtypes and cross-reactivity of asymptomatic positive, convalescent patients and negative individuals.** *A)* Detailed serum samples were analyzed for IgG, IgM, and IgA antibodies against SARS-CoV-2 Spike and RBD using ELISA. The groups were divided into negatives and positives based on the initial screening for anti-S IgG in asymptomatic individuals. *B)* Pie-charts showing the distribution of positive and COVID-19 samples for antibody sub-types of anti-S and anti-RBD antibodies. *C)* Serum antibodies against non-COVID coronavirus spike proteins analyzed by ELISA for IgG, IgM, and IgA in negatives (n=143), asymptomatic positives (n=348), and COVID-19 patients (n=94). Data are represented as the mean ± standard error of the mean. One-way ANOVA analyzed data with Tukey's multiple comparisons test. *p*-value *<0.05, **<0.01, ***<0.001, ****<0.0001.

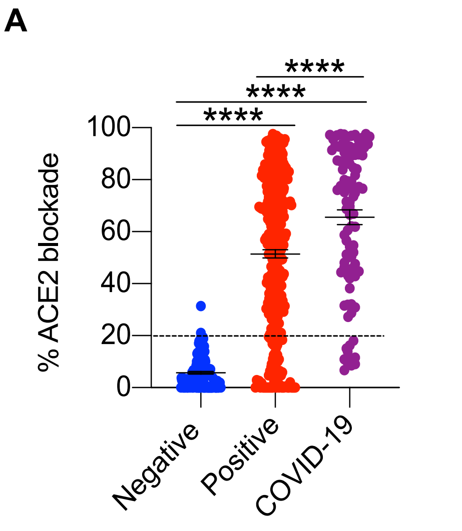

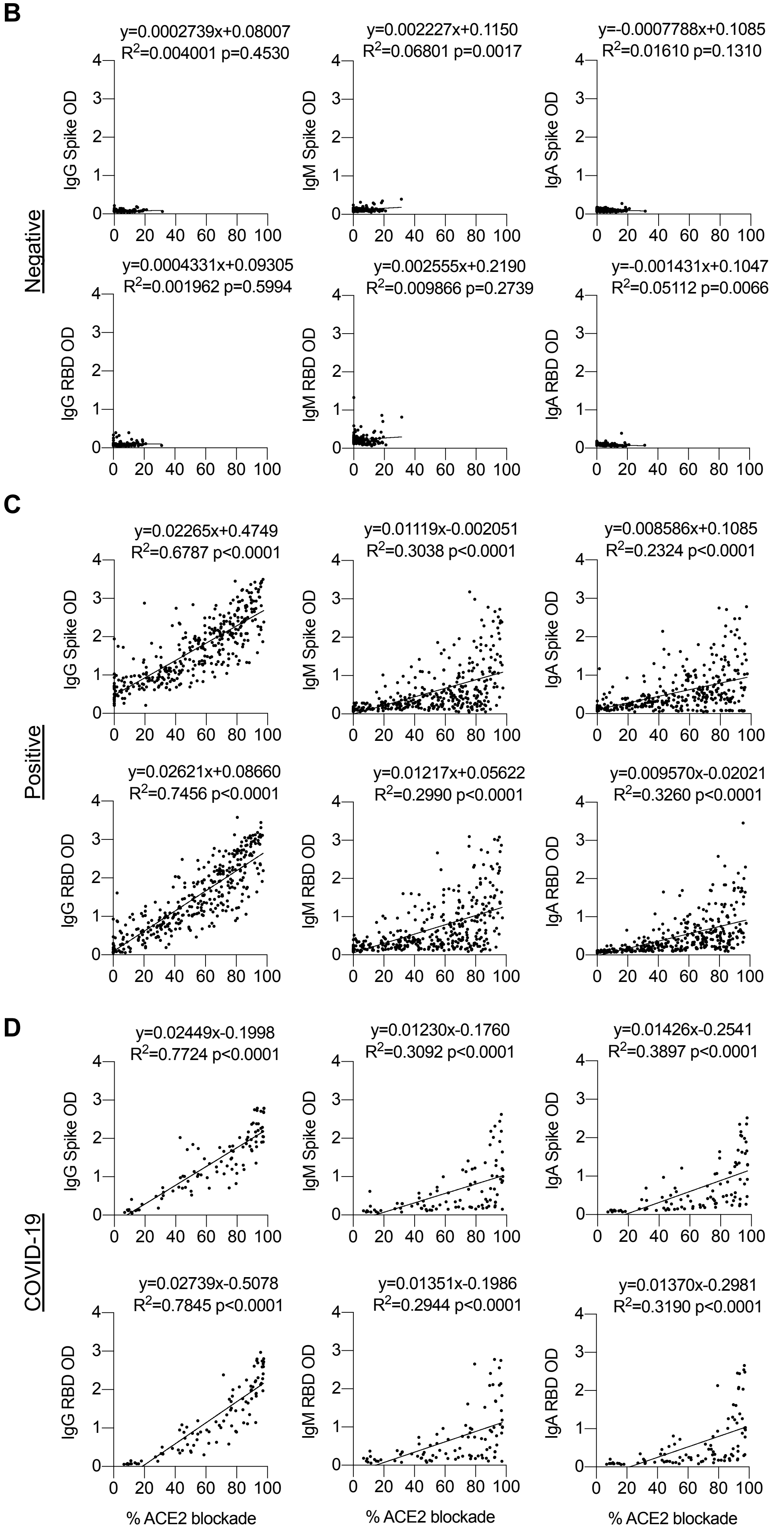

Figure S2. Related to Figure 3.* ACE2 binding inhibition in positive asymptomatic individuals and COVID-19 patients correlates with high anti-S/RBD IgG.** *A)* Serum inhibition of ACE2 binding from negative (n=143), asymptomatic positive (n=348), and COVID-19 patients (n=94) analyzed by ELISA. Data are represented as the mean ± standard error of the mean. One-way ANOVA with Tukey's multiple comparisons test was used to analyze inhibition of ACE2 binding. *p*-value *<0.05, **<0.01, ***<0.001, *<0.0001. *B)* Inhibition of ACE2 binding correlations against IgG, IgM, and IgA reactive to Spike or RBD. Data were analyzed by nonlinear regression and two-tailed correlation analysis.

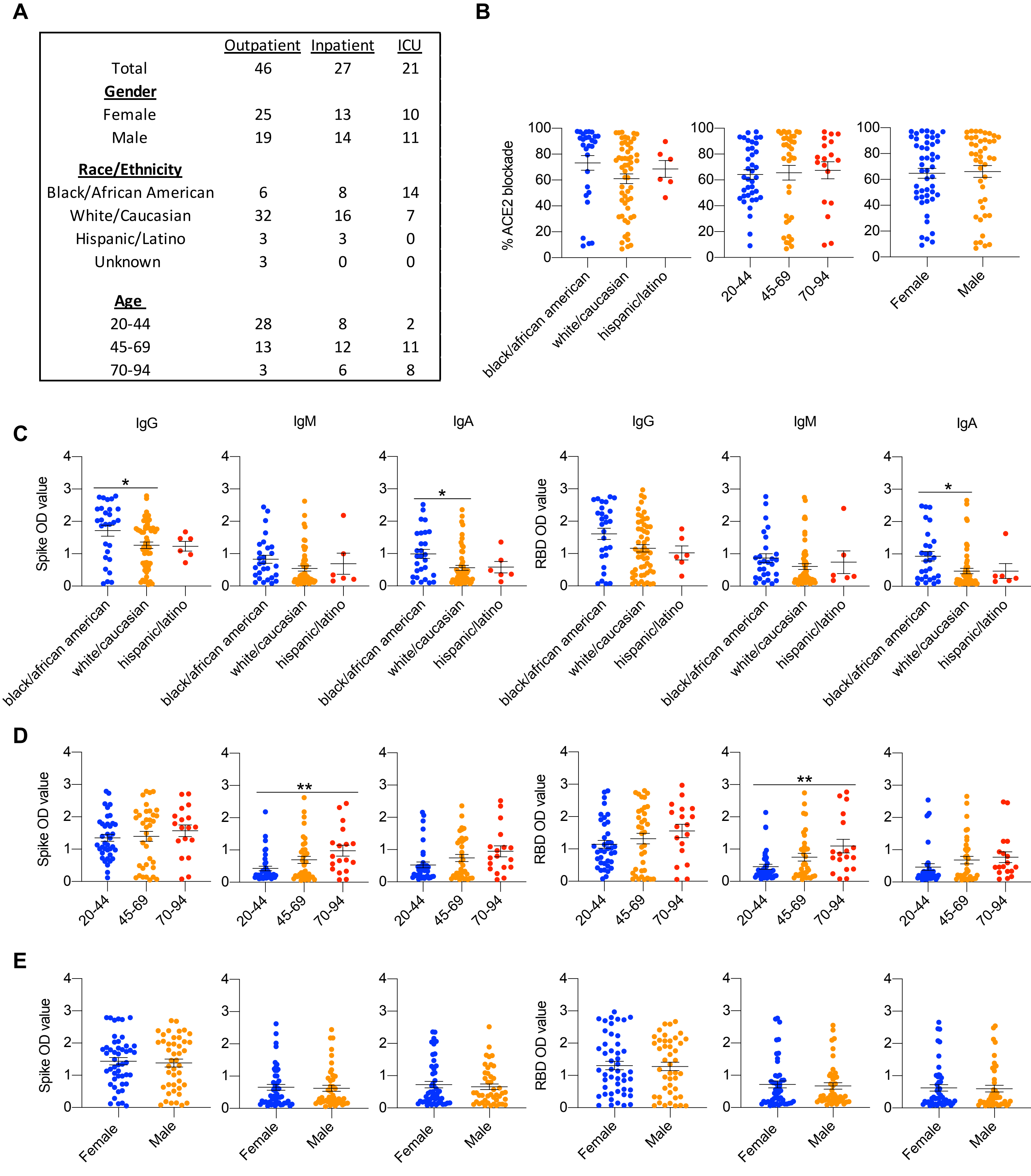

A

	Outpatient	Inpatient	ICU
Total	46	27	21
**Gender**			
Female	25	13	10
Male	19	14	11
**Race/Ethnicity**			
Black/African American	6	8	14
White/Caucasian	32	16	7
Hispanic/Latino	3	3	0
Unknown	3	0	0
**Age**			
20-44	28	8	2
45-69	13	12	11
70-94	3	6	8

Figure S3. Related to Figure 4. **COVID-19 patient antibody diversity and inhibition of ACE2 binding based on ethnicity, age, and sex.** Serum samples from COVID-19 positive patients were analyzed based on ethnicity, age, and sex for ACE2 binding inhibition, IgG, IgM, and IgA against Spike and RBD. *A)* Description of 94 patients in the clinical patient cohort. ELISA-based analysis of ACE2 binding inhibition *(B)* and IgG, IgM, and IgA against Spike and RBD based on ethnicity *(C)*, age *(D)*, and sex *(E)*. Data are represented as the mean ± standard error of the mean. One-way ANOVA analyzed data with Tukey's multiple comparisons tests or unpaired two-tail T-test for sex. *p*-value *<0.05, **<0.01, ***<0.001, ****<0.0001.

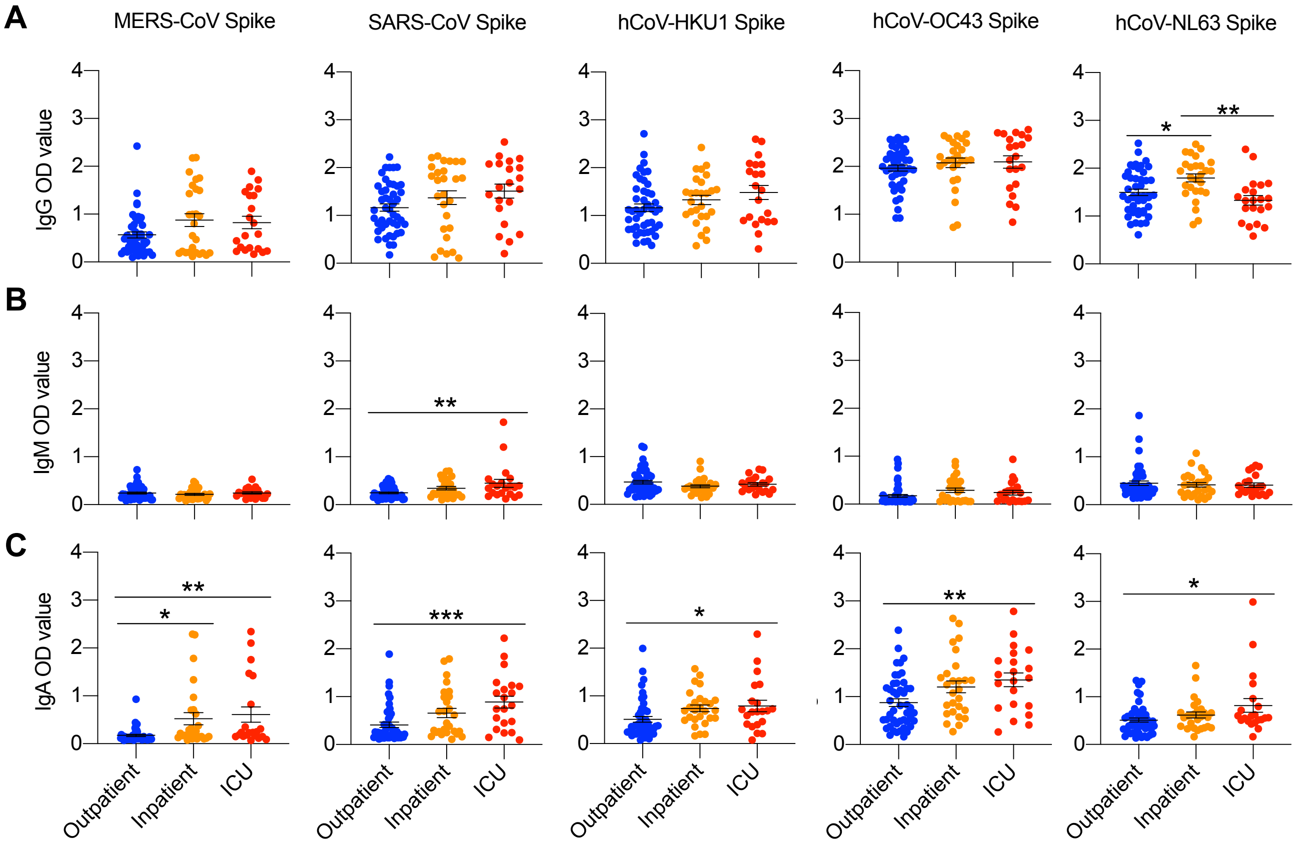

Figure S4. Related to Figure 4. **COVID-19 patient antibody subtypes and cross-reactivity.** Serum samples from COVID-19 positive individuals were analyzed for circulating antibodies against other related coronaviruses. ELISA-based analysis of serum antibodies against non-COVID coronavirus spike proteins for IgG *(A)*, IgM *(B)*, and IgA *(C)* in outpatient (n=46), inpatient (n=27), and ICU (n=21) patients. Data are represented as the mean ± standard error of the mean. One-way ANOVA analyzed data with Tukey's multiple comparisons tests. *p*-value *<0.05, **<0.01, ***<0.001, ****<0.0001.

	RBD		SPIKE	
RSV IgM 1	0.088	Negative	0.095	Negative
RSV IgM 2	0.072	Negative	0.121	Negative
RSV IgM 3	0.077	Negative	0.095	Negative
RSV IgM 4	0.068	Negative	0.077	Negative
RSV IgM 5	0.086	Negative	0.201	Negative
RSV IgG 1	0.075	Negative	0.088	Negative
RSV IgG 2	0.067	Negative	0.15	Negative
RSV IgG 3	0.059	Negative	0.104	Negative
RSV IgG 4	0.112	Negative	0.065	Negative
RSV IgG 5	0.104	Negative	0.069	Negative
HCV IgG 1	0.101	Negative	0.074	Negative
HCV IgG 2	0.061	Negative	0.089	Negative
HCV IgG 3	0.275	Negative	0.246	Negative
HCV IgG 4	0.225	Negative	0.098	Negative
HCV IgG 5	0.067	Negative	0.074	Negative
HCV IgM 1	0.095	Negative	0.184	Negative
HCV IgM 2	0.084	Negative	0.093	Negative
HCV IgM 3	0.064	Negative	0.072	Negative
HCV IgM 4	0.094	Negative	0.118	Negative
HCV IgM 5	0.081	Negative	0.108	Negative
ANA 1	0.076	Negative	0.155	Negative
ANA 2	0.075	Negative	0.067	Negative
ANA 3	0.079	Negative	0.064	Negative
ANA 4	0.092	Negative	0.08	Negative
ANA 5	0.101	Negative	0.076	Negative
HIV 1	0.07	Negative	0.096	Negative
HIV 2	0.079	Negative	0.17	Negative
HIV 3	0.096	Negative	0.075	Negative
HIV 4	0.19	Negative	0.121	Negative
HIV 5	0.36	Negative	0.196	Negative
HIV 6	0.114	Negative	0.13	Negative
HIV 7	0.081	Negative	0.068	Negative
HIV 8	0.083	Negative	0.105	Negative
HIV 9	0.108	Negative	0.082	Negative
HIV 10	0.08	Negative	0.085	Negative
OC43 1	0.068	Negative	0.082	Negative
OC43 2	0.072	Negative	0.128	Negative
OC43 3	0.121	Negative	0.181	Negative
OC43 4	0.107	Negative	0.098	Negative
OC43 5	0.098	Negative	0.091	Negative
NL63 1	0.074	Negative	0.08	Negative
NL63 2	0.089	Negative	0.148	Negative
NL63 3	0.07	Negative	0.07	Negative
NL63 4	0.192	Negative	0.159	Negative
**NL63 5**	**1.014**	**Positive**	**1.01**	**Positive**
**229E 1**	**1.095**	**Positive**	**1.117**	**Positive**
**229E 2**	**1.067**	**Positive**	**0.938**	**Positive**
229E 3	0.067	Negative	0.083	Negative
229E 4	0.079	Negative	0.091	Negative
229E 5	0.119	Negative	0.095	Negative
HKU1 1	0.112	Negative	0.09	Negative
HKU1 2	0.077	Negative	0.127	Negative
HKU1 3	0.097	Negative	0.087	Negative
HKU1 4	0.328	Negative	0.153	Negative
HKU1 5	0.083	Negative	0.107	Negative

Table S1. Related to STAR Methods. **SARS-CoV-2 ELISA validation for specificity.**
